# Recent Progress on Two-Dimensional Nanoflake Ensembles for Energy Storage Applications

**DOI:** 10.1007/s40820-018-0219-z

**Published:** 2018-08-20

**Authors:** Huicong Xia, Qun Xu, Jianan Zhang

**Affiliations:** College of Materials Science and Engineering, Zhengzhou University, Zhengzhou, 450001 People’s Republic of China

**Keywords:** 2D nanoflakes, Ensembles, 3D architectures, Supercapacitors, Lithium-ion batteries, Sodium-ion batteries

## Abstract

The rational design and synthesis of two-dimensional (2D) nanoflake ensemble-based materials have garnered great attention owing to the properties of the components of these materials, such as high mechanical flexibility, high specific surface area, numerous active sites, chemical stability, and superior electrical and thermal conductivity. These properties render the 2D ensembles great choices as alternative electrode materials for electrochemical energy storage systems. More recently, recognition of the numerous advantages of these 2D ensemble structures has led to the realization that the performance of certain devices could be significantly enhanced by utilizing three-dimensional (3D) architectures that can furnish an increased number of active sites. The present review summarizes the recent progress in 2D ensemble-based materials for energy storage applications, including supercapacitors, lithium-ion batteries, and sodium-ion batteries. Further, perspectives relating to the challenges and opportunities in this promising research area are discussed.
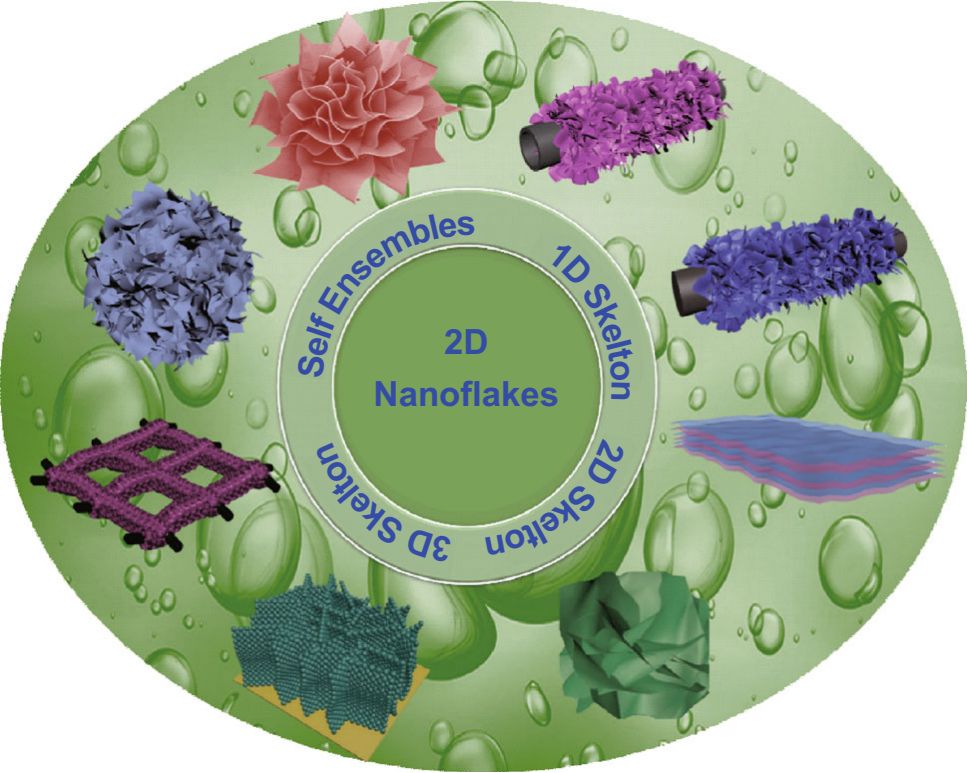

## Highlights

- In this review, we emphasize the recent developments on two-dimensional nanoflake ensembles and their applications for enhanced electrochemical performance in supercapacitors, lithium-ion batteries, sodium-ion batteries, potassium-ion batteries, and zinc-ion batteries.
- An overview of recent advances in three-dimensional hierarchical structures from two-dimensional nanoflake ensembles with controllable shape and compositions is provided.
- Enhanced electrochemical energy storage performance based on assemblies of these two-dimensional nanoflake ensembles is discussed in detail.

## Introduction

**Fig. 1:**
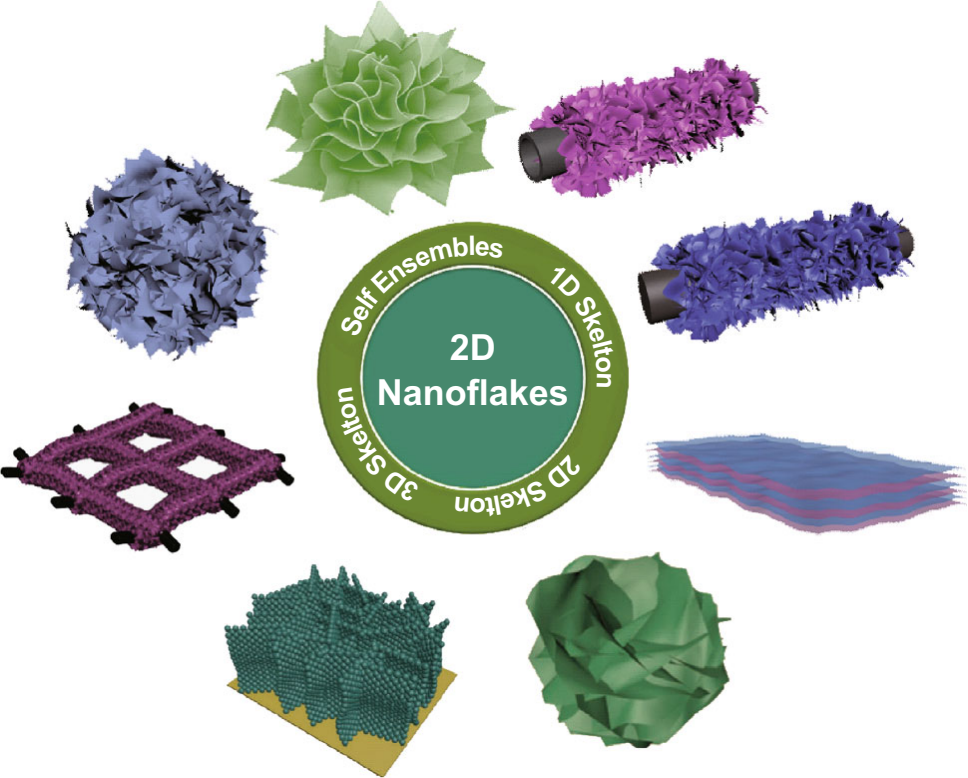
Schematic illustration of 3D architecture-based 2D nanoflake ensembles

Two-dimensional (2D) nanoflake-based materials were predicted to be intrinsically unstable until 2004 when graphene was successfully synthesized [1, 2]. The discovery of 2D nanoflake-based materials has attracted much interest due to the prospects of these materials for advanced energy storage systems [3–5]. Energy storage has become a global concern due to the growing energy demand; thus, two-dimensional nanoflake-based materials have attracted increasing attention for many energy-related applications [6–9]. Based on their unique properties, 2D nanoflake ensemble-based materials are expected to play more important roles in energy storage devices [10–12]. Two-dimensional nanoflake ensemble-based materials with different geometrical features have been intensively investigated in recent years (Fig. 1) [13–15]. Due to their lower dimension, these structures should have large specific surface areas and more active sites, and the surface/interface states become more essential and even dominant compared to normal three-dimensional (3D) nanomaterials [16]. Furthermore, these 2D nanoflake ensemble-based materials provide enhanced mechanical properties, large specific surface area, and rapid mass and electron transport kinetics due to the combination of the excellent connatural properties of the 2D nanoflake materials and the unique 3D structures of the ensembles [17, 18]. The large surface area provides the capacity for guest ion intercalation, fast ion diffusion, and speedy charge transfer along the channels. Scientists have paid much attention to 2D nanoflake ensemble-based materials with few or atomic layer thickness, and the number of related publications has increased sharply [19]. The ensembles of 2D nanoflake materials form 3D nanostructures based on either physical or chemical interlinking. Two-dimensional nanoflake ensemble-based materials have attracted great research interest and have wide potential for application in physics, chemistry, and materials science. With regard to their unique structural features of high specific surface area, elasticity, and chemical stability, 2D nanoflake ensemble-based materials have been used as building blocks for 3D hierarchical materials with desirable functionality [20]. To broaden the scope for use in renewable energy, efficient energy storage technologies are required. Among these applications, portable/hybrid electric vehicles have been developed to the meet needs of our modern information-rich and mobile society.

Although this article summarizes many of the historically significant studies on 2D nanoflakes, the most recent developments are emphasized. Notably, this article aims to describe the different processes used for the preparation of 2D nanoflake-based 3D architectures and the different areas of application in which the unique structures of these materials can be harnessed to achieve greater efficacy as compared to their 2D analogues, and even provide avenues for different applications. Electrochemical systems for energy storage, including supercapacitors, lithium-ion batteries, sodium-ion batteries, potassium-ion batteries, and zinc-ion batteries for advanced energy storage devices, are also discussed. Finally, the conclusions and prospective for these emerging composites are discussed.

Three-dimensional architectures composed of 2D nanoflakes generally possess a large surface area. Likewise, these unique 3D structures can better provide electronic transport and ion transfer channels and increase the contact area between the electrolyte and the active material because of the larger specific surface area. These features are beneficial for achieving improved electrochemical reaction kinetics. Moreover, the 3D structures assembled from these 2D nanoflakes exhibit good electrochemical properties. Thus, the characteristics of the 2D units can be retained in the 3D structure, leading to more extensive applications. Moreover, the deficiencies of the 2D nanoflake materials can be offset in these 3D materials, where the 2D nanoflake materials are connected to the 3D mesh material, thereby improving the scope of application.

## Synthesis of 2D Nanoflake Ensemble-Based 3D Nanostructures

Two-dimensional nanoflakes, compared to spherical/bulk structures of the same volume, could contribute much more to achieving higher viscosity in their stretched state, and the channeled network generated by these 2D nanoflakes can also form ensembles to furnish 3D hierarchical nanostructures [21]. Two-dimensional nanoflake-based 3D hierarchical frameworks have been prepared by self-assembly into ensembles. Although the first step is to prepare 2D nanoflakes, all the current methods can be classified into two categories: top-down approaches and bottom-up approaches, including mechanical cleavage, liquid exfoliation, ion intercalation and exfoliation, selective etching and exfoliation, chemical vapor deposition (CVD), and wet-chemical synthesis [22–25]. In the assembly of 2D nanoflakes into 3D hierarchical structures, the unique characteristics of the individual 2D nanoflakes can be largely maintained, and these structures have thus recently attracted great interest for fundamental investigations and practical applications in diverse technologies.

Gong et al. [26] constructed 3D architectures from 2D nanoflake building blocks such as MoS_2_ and graphene oxide nanoflakes by exploiting their controllable assembly character. The MoS_2_ nanoflakes were first fabricated via liquid exfoliation, and graphene oxide was produced by the modified Hummer’s method. The prepared MoS_2_ and graphene oxide nanoflakes were then employed as building blocks for assembly via hydrothermal processing. Finally, the graphene oxide in the resulting samples was chemically reduced to graphene, giving rise to MoS_2_-graphene architectures with different MoS_2_ contents. The as-prepared MoS_2_-graphene architectures possess a 3D structure with interconnected pores ranging from several nanometers to several micrometers. The sectional overlapping or connection of the flexible nanoflakes might be the result of cross-linking of the functional groups in the graphene nanoflakes, similar to those of pure graphene oxide and graphene hydrogels.

In addition to the aforementioned 2D nanomaterials, there are many other kinds of 2D nanostructured materials, such as MXenes, covalent organic frameworks (COFs), 2D polymers, metal organic frameworks (MOFs), and 2D supramolecular organic nanostructures. MXenes, a novel family of 2D metal carbides, have demonstrated potential as electrode materials for energy storage devices with a volumetric capacitance exceeding that of all-carbon materials [27–31]. Liu et al. [32] reported simple ensembles of transition metal oxide (TMO) nanostructures (including TiO_2_ nanorods and SnO_2_ nanowires) on MXene (Ti_3_C_2_) nanoflakes, assembled through van der Waals interactions. The MXene nanoflakes, acting as the underlying substrate, not only enabled reversible transport of electrons and ions at the interface, but also prevented aggregation of the TMO nanostructures during lithiation/delithiation. Specifically, in the SnO_2_ nanowires, which are notorious for severe volume expansion, the MXene nanoflakes could alleviate pulverization by providing excellent mechanical flexibility. More importantly, TMOs can confer extraordinary electrochemical properties to composites, and their nanoscale size offers short lithium diffusion pathways and additional active sites. Two-dimensional COF nanoflakes, in which the molecular building blocks form robust microporous networks, are accommodative of Li salts and are considered as potential candidates for solid-state fast Li^+^ conductors [33–39]. Chen et al. [40] demonstrated the first example of cationic moieties incorporated into the skeleton of a COF material that could indeed split the ion pair of the Li salt, increase the concentration of free mobile Li^+^, and thus improve the Li^+^ conductivity in COF-based Li^+^ conductors. Two-dimensional COF nanoflakes were selected as the COF matrix for this study due to their high surface area and unique 2D framework, both of which are beneficial for exposing the ionic moieties to the Li salts.

## 2D Nanoflake Ensemble-Based Materials for Supercapacitors

Supercapacitors have received widespread attention due to their long cycle life, high-power density, environmental friendliness, and rapid charge and discharge ability [41–43]. Three-dimensional structures composed of 2D nanoflakes have been used as electrode materials for electric double-layer capacitors and pseudo-capacitors [44–46]. The capacity stems from surface ion adsorption, surface redox reactions, and fast ion intercalation without phase change [47–49]. Due to their unique structure and excellent electrochemical properties, these 3D structures composed of 2D nanoflakes have received much attention. For example, 2D graphene nanoflakes easily agglomerate under an external force, which negatively affects the inherent properties of the nanoflakes, such as by decreasing the specific surface area, which eventually leads to a decrease in the energy storage performance [50–52]. In the process of assembling a 3D structure from 2D nanoflakes, the advantages of the original 2D nanoflakes can be well preserved, which has fueled a great deal of basic scientific research and interest in numerous nanotechnologies. Here, we summarize some reports on three-dimensional structures comprising two-dimensional nanoflakes and their application to supercapacitor devices. The composites not only exhibit the characteristics of the 2D nanoflake materials, but also exhibit unique characteristics of the 3D structure, thereby furnishing high specific surface area, rapid ion transport and electron conduction, and excellent stability. In supercapacitor applications, these materials give rise to excellent rate performance, super-high capacitance, and remarkable cyclic stability.

### Carbon Nanoflakes

**Fig. 2:**
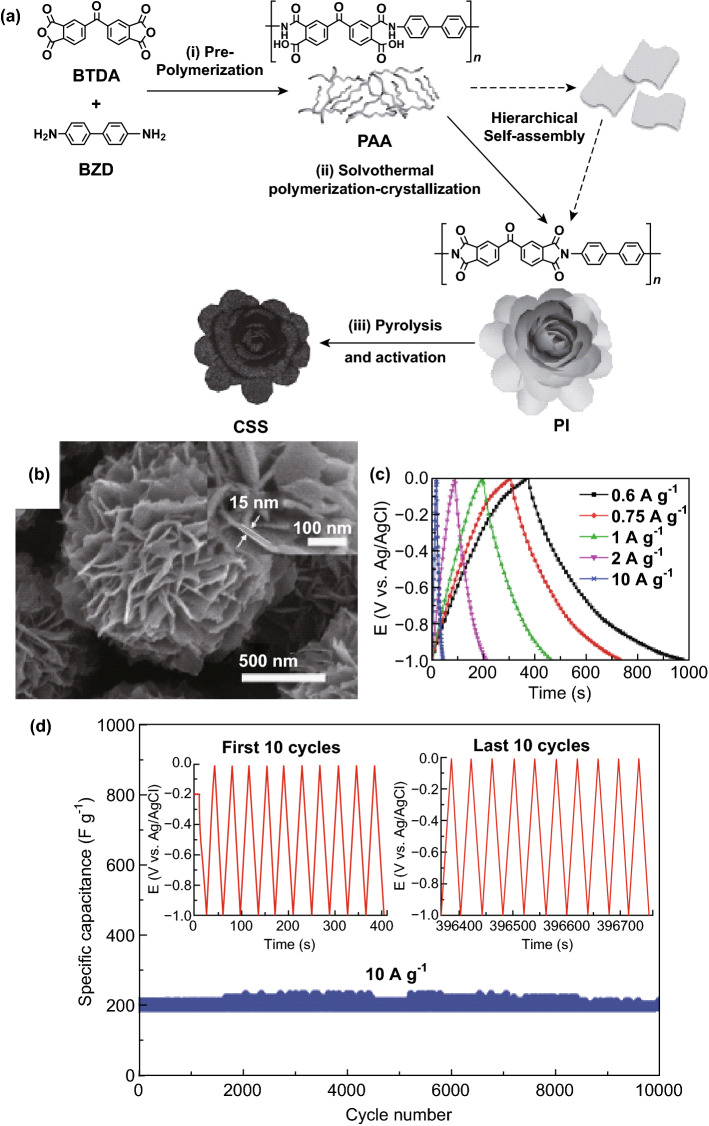
3D architectures based on 2D carbon nanoflake ensembles for supercapacitors. **a** Overall fabrication of 3D carbon superstructures. **b** SEM images of carbon materials with various hierarchical structures, derived from polyimide. **c** Galvanostatic charge/discharge curves at various current densities and **d** cycling performance at a current density of 10 A g^−1^, indicating excellent cycling stability of N-doped porous carbons. Reproduced with permission [56]. Copyright ©2016, John Wiley & Sons, Inc.

In the last decade, increasing attention has been paid to 3D structures comprising low-dimensional materials such as one-dimensional (1D) carbon nanotubes and 2D graphene nanoflakes [53–55]. These structures have the characteristics of the constituent units as well as some unexpected advantages, which make them excellent choices for energy storage materials. The supercapacitor performance of 3D ensembles of carbon networks has been studied in detail. Xu and co-workers developed 3D carbon superstructures with fine-tunable nanoflakes through the hierarchical assembly of polyimide, for use as electrode materials in supercapacitors (Fig. 2) [56]. The specific capacitance was as high as 364 F g^−1^ at 0.6 A g^−1^, where 55% of the current density was maintained with a change from 0.6 to 10 A g^−1^. The excellent rate performance is due to the large number of pores in the 3D structure composed of 2D carbon nanoflakes and the contact area between the electrolyte and active substances, which allowed rapid mass transport of ions during the charge and discharge process. On the other hand, the 2D nanoflakes, as the primary component of the 3D nanoarchitectures, provided adequately exposed electroactive sites for catalysis of the reactions and charge storage and served as a long-distance in-plane charge transporter. The 3D carbon superstructures also displayed robust cycling stability (200 F g^−1^ at 10 A g^−1^, even after 10,000 cycles). Similarly, improved capacitance was observed for other composite 3D graphene assemblies. Li et al. [57] designed and fabricated holey graphene oxide (GO) ensembles into a 3D interconnected network with hierarchical holes in the graphene layers, where the constituents provided strength and furnished more edge active sites and more ion channels, leading to better electrochemical behavior and a specific capacitance of 219.6 F g^−1^ at 1 A g^−1^. The three-dimensional graphene/nanostructured conductive polymer hydrogel supercapacitor could be reversibly cycled at the potential of 0.8 V and displayed a high volumetric energy density of 8.80 mWh cm^−3^ at a power density of 30.77 mW cm^−3^ [58]. Furthermore, superior cycling stability, with 86% capacitance retention, was achieved after 17,000 cycles. Compared with similar materials previously reported, this composite exhibited excellent electrochemical performance. Moreover, its excellent performance satisfies the requirements of electric vehicles, which should greatly enhance the range of applications of related supercapacitors.

### Transition Metal-Based Nanoflakes

**Fig. 3:**
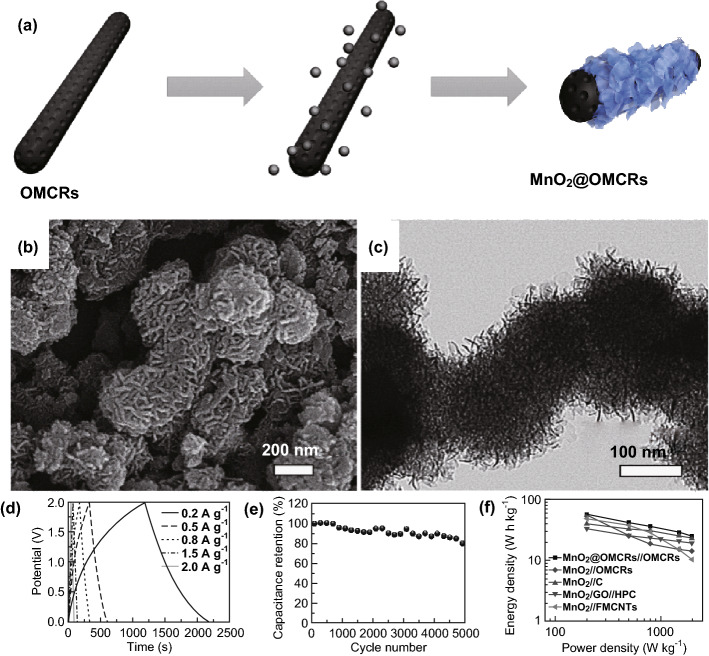
3D architectures based on 2D MnO_2_ nanoflake ensembles for supercapacitors. **a** Schematic illustration of the process for synthesis of MnO_2_@-ordered mesoporous carbon nanorods. **b** SEM and **c** TEM images of MnO_2_@-ordered mesoporous carbon nanorods. **d** Charge/discharge curves of asymmetric supercapacitor device at different current densities. **e** Capacitance retention as a function of cycle number at a current density of 2 A g^−1^. **f** Ragone plots of the present asymmetric supercapacitor devices and other reported MnO_2_-based asymmetric supercapacitor devices. Reproduced with permission [69]. Copyright ©2016, John Wiley & Sons, Inc.

**Fig. 4:**
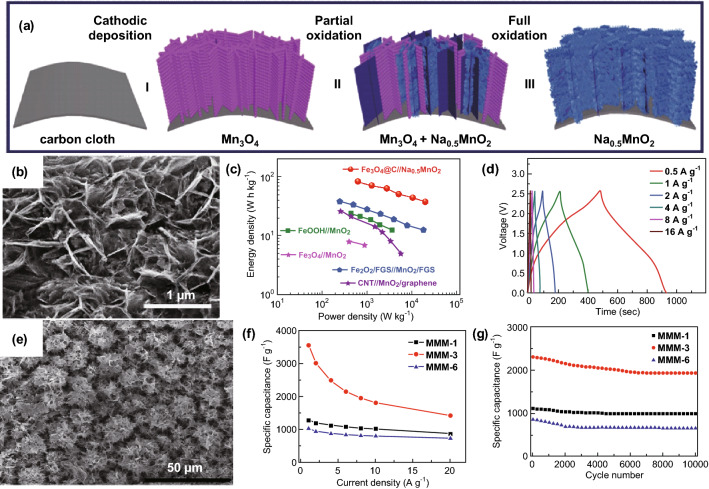
3D architectures based on 2D transition metal nanoflakes and 3D skeleton ensembles for supercapacitors. **a** Schematic illustration of morphological evolution from Mn_3_O_4_ nanowall arrays to Na_0.5_MnO_2_ nanowall arrays through electrochemical oxidation. **b** FESEM images of Na_0.5_MnO_2_ nanowall arrays. **c** Ragone plots for Na_0.5_MnO_2_//Fe_3_O_4_@C ASC and asymmetric supercapacitors reported in the literature. **d** Charge/discharge curves for Na_0.5_MnO_2_//Fe_3_O_4_@C asymmetric supercapacitors between 0 and 2.6 V at different current densities. Reproduced with permission [70]. Copyright ©2017, John Wiley & Sons, Inc. **e** SEM images showing the surface morphologies of nickel foam@rGO/Ag/Ni_3_S_2_ composite. Reproduced with permission [71]. Copyright ©2017, John Wiley & Sons, Inc. **f** Specific capacitance of MnMoO_4_·H_2_O@MnO_2_-1, MnMoO_4_·H_2_O@MnO_2_-3, and MnMoO_4_·H_2_O@MnO_2_-6 electrodes as a function of current density. **g** Corresponding cycling test at the same current density of 5 A g^−1^. Reproduced with permission [72]. Copyright ©2016, Elsevier

With the increasing number of reports on 3D structural materials, 3D structures composed of 2D transition metal nanoflakes have attracted much attention [59–62]. A large number of 3D structures composed of 2D transition metal nanoflakes have been developed and prepared. Previously, Xia and co-workers reported 2D nanoflake-assembled spherical microstructures as supercapacitor electrodes [63]. Significantly, in the 3D network structure formed by interconnection and assembly of the 2D transition metal nanoflakes, the reduced distance for electron transport improved the resistance to volume change caused by ion insertion and release and ultimately improved the performance of the supercapacitors. The synergistic effect of the dual metallic ions in Ni/Co-MOF nanoflakes enhanced the reactivity during energy storage, thereby enhancing electron and charge transport and accelerating the reaction kinetics. Moreover, the Ni/Co-MOF nanoflakes were assembled in a layer-by-layer architecture, which prevented coalescence of the nanoflakes (enhancing the stability). To achieve a higher energy density without sacrificing the power density, two important issues should be addressed: (1) the preparation of high-performance electrode materials as the first choice for supercapacitors; (2) the assembly of various types of supercapacitors [64]. Among the various materials that have been reported, manganese dioxide (MnO_2_) is the best choice for electrode materials due to its high theoretical specific capacity, low economic cost, and environmental friendliness [65–68]. However, the poor conductivity of MnO_2_ leads to unsatisfactory rate performance. Shang and co-workers used 2D MnO_2_ nanoflakes on the surface of 1D-ordered mesoporous carbon nanorods as a hybrid electrode for enhancing the performance of asymmetric supercapacitors (Fig. 3) [69]. The optimized nanocomposite displayed a maximum specific capacitance of up to 100 F g^−1^ at 0.2 A g^−1^ and a high energy density of 55.2 Wh kg^−1^ at a power density of 200.2 W kg^−1^ within a wide operating voltage of 2.0 V. In addition, direct self-assembly of 2D nanoflakes and carbon cloth was carried out for the fabrication of high-performance asymmetric supercapacitors that displayed excellent rate performance between 0 and 2.6 V and could still deliver a large specific capacitance of 88 F g^−1^ at a current density of 0.5 A g^−1^ and deliver a maximum energy density of about 81 Wh kg^−1^ at a power density of 647 W kg^−1^ (Fig. 4a–d) [70]. In addition, superior cycling stability with 96% capacitance retention was achieved after 10,000 cycles at a current density of 4 A g^−1^. Qi et al. [71] reported a 3D bush structure of Ag nanoparticle-decorated Ni_3_S_2_ grown on reduced graphene oxide (rGO) for high-performance supercapacitor electrodes, which displayed a record-high specific capacitance, excellent rate capability, and improved cycling stability compared with all the reported Ni_3_S_2_ electrode materials (Fig. 4e). The energy density achieved with the rGO/Ag/Ni_3_S_2_ nanocomposites was 28.7 Wh kg^−1^ at a power density of 425 W kg^−1^, and a value of 20.2 Wh kg^−1^ was maintained at a power density of 6799.3 W kg^−1^. Xu et al. [72] reported hierarchical MnMoO_4_·H_2_O@MnO_2_ nanoflake arrays on nickel foam for asymmetric supercapacitors (Fig. 4f–g). The fabricated core–shell structured asymmetric supercapacitor with MnMoO_4_·H_2_O@MnO_2_ nanocomposites was found to have the advantages of high capacitance (3560.2 F g^−1^ at 1 A g^−1^), superior flexibility, and high energy and power density (45.6 Wh kg^−1^ at 507.3 W kg^−1^). The resulting supercapacitors could operate at a high rate, even up to 20 A g^−1^, exhibited excellent cycle life, retained 84.1% of the original capacitance after cycling for 10,000 loops, and exhibited amazing flexibility.

The electrochemical performance of 2D nanoflake ensemble-based composites, when used as electrodes and tested under different conditions for supercapacitors, is compared and summarized in Table 1. Because these 2D nanoflakes assembled into 3D materials have good redox properties, they generally exhibit excellent supercapacitor performance. For future development of supercapacitors, the challenge is to better assemble the 2D nanoflakes while maintaining the original characteristics of the building blocks and controlling the characteristics of the synthesized 3D materials.

**Table 1:** Comparison of electrochemical performance of 2D nanoflake ensemble-based materials for supercapacitors

Materials	Rate performance	Cycling performance	Refs.
Ni/Co-MOF nanoflakes	530.4 F g^−1^ at 0.5 A g^−1^	180 F g^−1^ after 2000 cycles at 2 A g^−1^	[63]
3D carbon superstructures	364 F g^−1^ at 0.6 A g^−1^	200 F g^−1^ after 10,000 cycles at 1 A g^−1^	[56]
MnO_2_@OMCRs	99.5 F g^−1^ at 0.2 A g^−1^	39.9 F g^−1^ after 5000 cycles at 2 A g^−1^	[69]
Na_0.5_MnO_2_ nanoflakes-assembled nanowall arrays	366 F g^−1^ at 1 A g^−1^	300 F g^−1^ after 10,000 cycles at 4 A g^−1^	[70]
PANI/rGO	112 F g^−1^ at 0.08 A g^−1^	51.6 F g^−1^ after 17,000 cycles at 1.26 A g^−1^	[58]
rGO/Ag/Ni_3_S_2_	5920 mF cm^−2^ at 5 mA cm^−2^	4012.2 mF cm^−2^ after 3000 cycles at 30 mA cm^−2^	[71]
Hierarchical MnMoO_4_·H_2_O@MnO_2_ core–shell nanoflakes	3560.2 F g^−1^ at 1 A g^−1^	2994.2 F g^−1^ after 10,000 cycles at 1 A g^−1^	[72]
Nickel sulfides/MoS_2_	757 F g^−1^ at 0.5 A g^−1^	445.2 F g^−1^ after 2000 cycles at 5 A g^−1^	[73]

## 2D Nanoflake Ensemble-Based Materials for Lithium-Ion Batteries

Lithium-ion batteries (LIBs) are currently the greenest power source with chargeability and are widely used in daily life [73–76]. Similar to 2D nanoflakes (e.g., transition metal dichalcogenides and metal oxides), 3D architectures have been used as active electrode materials in LIBs [77–79]. In order to meet the ever-increasing energy demand, the preparation of new synthetic electrode materials is imperative. Due to the huge volume change of electrode materials during the lithiation process, it is still a challenge to find suitable electrode materials.

### Transition Metal-Based Nanoflakes

**Fig. 5:**
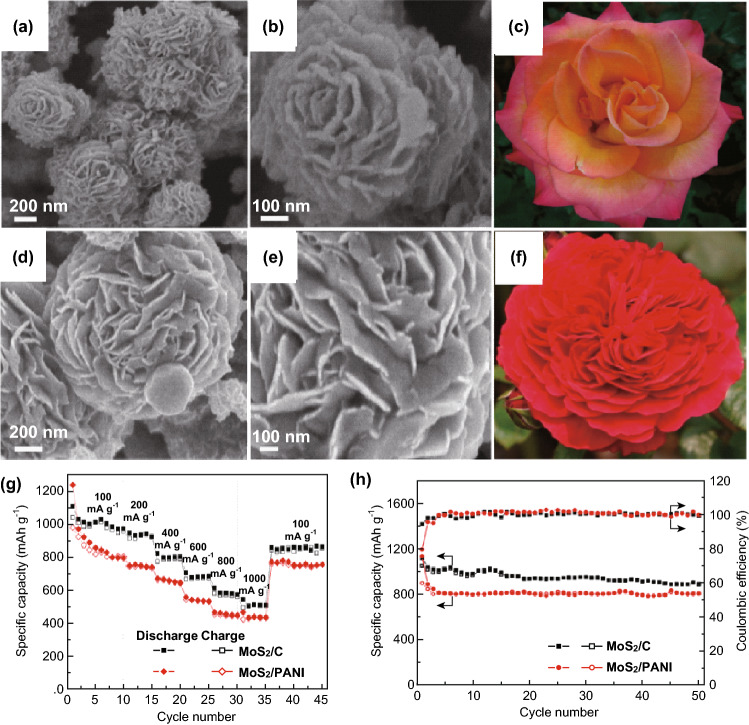
3D architectures based on 2D transition metal nanoflake self-assemblies for LIBs. SEM images of 3D hierarchical MoS_2_/PANI (**a**, **b**) and MoS_2_/C (**d**, **e**) nanoflowers. Photographs of two types of Chinese roses (**c**, **f**). **g** Cycling performance and Coulombic efficiency of 3D hierarchical MoS_2_/PANI and MoS_2_/C nanoflowers measured in the voltage range of 0.005–3.0 V at a current density of 100 mA g^−1^. **h** Rate capability of 3D hierarchical MoS_2_/PANI and MoS_2_/C nanoflowers between 0.005 and 3 V at different current densities. Reproduced with permission [89]. Copyright ©2014, American Chemical Society

Compared with the corresponding bulk materials, transition metal-based nanoflakes have a higher specific surface area and better electrochemical performance because of the size effect [80–82]. This has inspired a plethora of studies on the design of various nanoflakes in attempts to modify their properties by controlling the morphologies and sizes [83–85]. Carbon-coated MoS_2_ flower-like nanostructures were used as electrode materials and exhibited greatly enhanced electrochemical properties compared to the MoS_2_ nanoflakes, including notably high reversible capacities (1419 mAh g^−1^ and 885 mAh g^−1^ at 0.1 A g^−1^), outstanding rate capabilities (672 and 450 mAh g^−1^ at 10.0 A g^−1^), and excellent cycling stability (80% capacity retention after 50 cycles at 0.1 A g^−1^) [86]. The 2D MoS_2_ nanoflakes gave rise to a large specific surface area and increased interlayer distance, leading to a shorter diffusion path for Li^+^ and rapid electron transport [87, 88]. The defects in the pure 3D hierarchical materials limited achievement of the theoretical capacity and lowered the rate performance. Hu et al. fabricated 3D hierarchical MoS_2_/polyaniline nanoflowers with high reversible capacity and excellent rate capability for LIBs (Fig. 5) [89]. It was found that the combination of the high-capacity active materials and 2D nanoflake ensemble-based materials could provide favorable diffusion kinetics for both electrons and Li^+^ via the numerous open channels and multi-dimensional electronic networks [90]. Additionally, the specific capacity and rate capability were highly enhanced due to the building blocks with fully exposed active edges oriented in a preferred manner [91]. Chen et al. [92] used Mo-based ultrasmall nanoparticles incorporated into carbon nanoflakes to achieve high and stable electrochemical performance for lithium-ion storage. The ultrasmall nanoparticles completely confined the redox reaction in their surface region, thereby maximizing utilization of the active materials. Moreover, the incorporated nanostructures effectively prevented the formation of MoO_2_ atomic clusters and Mo_2_C nanocrystals via coalescence or oxidization, endowing the composites with rapid electron transfer, which resulted in compact Li storage with high-power performance. Zhang et al. [93] synthesized metal and Se co-doped NbS_2_ nanoflakes by a facile oil-phase synthetic process and effective self-assembly strategy. These materials exhibited unprecedented fast surface-controlled lithium storage behavior, rather than the conventional slow diffusion-controlled mechanisms encountered in most battery materials.

Molybdenum sulfide (MoS_2_) has a high theoretical specific capacity (670 mAh g^−1^), excellent rate performance, and satisfactory cycle stability [94]. Because MoS_2_ is formed by weak van der Waals forces, the volume effect in the process of ion intercalation is smaller, and the integrity of the electrode material structure is better ensured [95]. However, the further application of MoS_2_ is hindered by its poor electrical conductivity and unsatisfactory rate performance. Thus, in attempts to solve these problems, recent remarkable advances have involved the engineering of novel MoS_2_/N-doped graphene nanoflakes with highly improved capacity, rate capability, and stability for LIBs by the invention/introduction of a MoS_2_/N-doped graphene interface [96, 97]. The strategy of doping carbon nanoflakes can adequately enhance electron transfer and ion diffusion. However, this improvement is limited by the limited contact sites of the carbon nanoflakes and MoS_2_.

### Transition Metal-Based Nanoflakes on 1D Skeleton

Transition metal-based nanoflake electrodes still face great challenges for practical application, such as fast capacity fading caused by the drastic volume change upon cycling and inferior rate performance due to the poor conductivity. The novel structure composed of ultrathin SnO_2_ nanoflakes with smaller plate-like units can shorten the distance between ions and electrons, increase the reaction kinetics, and increase the area of contact between the active material and the electrolyte, leading to optimized rate capability, and the low-dimensional tubular hollow structure can alleviate the volume change caused by ion insertion and structural collapse, thereby furnishing improved lithium storage [98]. For instance, Xia et al. demonstrated an unexpected result in which a 1D Cu(OH)_2_ nanorod/2D SnO_2_ nanoflake core/shell heterostructure covered with a graphene layer with internal void spaces could be facilely prepared by an in situ growth strategy [99]. It was found that the open space provided a larger reaction surface area and allowed for diffusion of the electrolyte into the inner region of the electrode; thus, the nanoflakes could provide enough sites for insertion and extraction of lithium ions and could alleviate the problems of agglomeration and crushing of the active materials due to volume changes. The unique 3D structure comprising 2D nanoflakes was effective for resolving these problems.

**Fig. 6:**
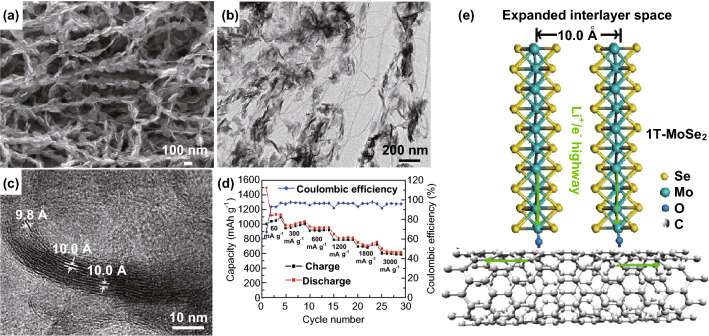
3D architectures based on 2D MoSe_2_ nanoflakes on 1D SWCNT skeleton for LIBs. **a** SEM image and **b** TEM image of the hybrids, revealing MoSe_2_ nanoflakes anchored intimately on the surface of a SWCNT bundle. **c** Corresponding HRTEM image showing enlarged interlayer distance of 10.0 Å. **d** Rate performance of 1T-MoSe_2_/SWCNTs hybrids. **e** Schematic illustration showing the electrochemical process in 1T-MoSe_2_/SWCNT electrode. Reproduced with permission [100]. Copyright ©2017, American Chemical Society

**Fig. 7:**
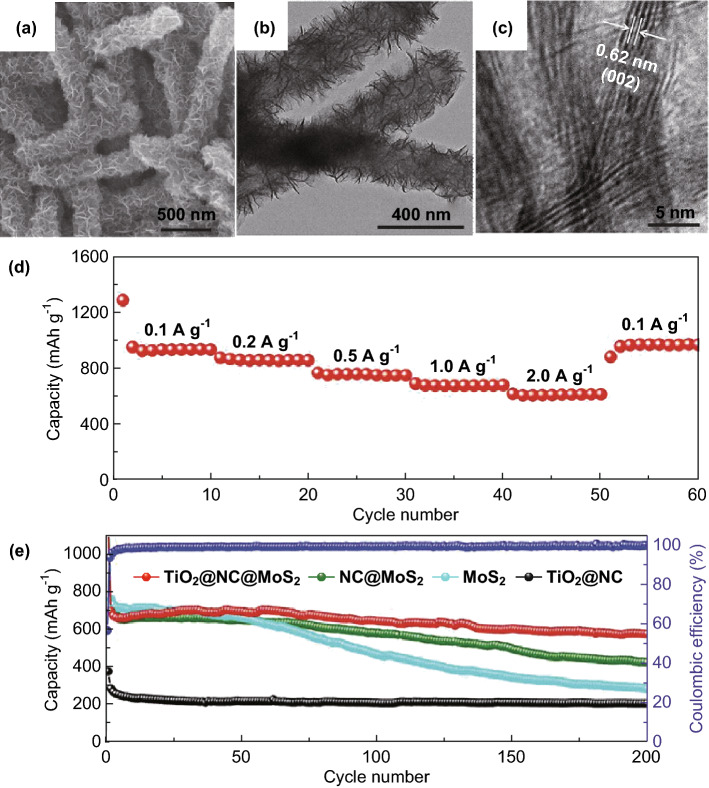
3D architectures based on 1D TiO_2_ nanotubes@carbon layer@2D MoS_2_ nanoflakes for LIBs. **a** SEM and **b** TEM images of annealed TiO_2_@NC@MoS_2_ nanotubes. **c** HRTEM image of MoS_2_ nanoflakes. **d** Rate performance at various current densities. **e** Cycling performance of TiO_2_@NC@MoS_2_, NC@MoS_2_, MoS_2_, and TiO_2_@NC at a current density of 1.0 A g^−1^ and the corresponding Coulombic efficiency of TiO_2_@NC@MoS_2_. Reproduced with permission [102]. Copyright ©2017, John Wiley & Sons, Inc.

Xiang et al. [100] successfully developed metallic 1T-MoSe_2_ nanoflakes with an expanded interlayer distance, embedded on single-walled carbon nanotubes (SWCNTs), for improving LIBs, where the unique architecture provided an expanded interlayer spacing of 10.0 Å and the abundant active edge sites of the 1T-MoSe_2_ nanoflakes promoted diffusion of the lithium ions and penetration of the electrolyte (Fig. 6). The highly conductive SWCNTs and the metallic conductivity of the 1T-MoSe_2_ constructing the electron/ion transfer channel led to a remarkable rate performance of 630 mAh g^−1^, even at 3 A g^−1^. Furthermore, density functional theory (DFT) calculations indicated that the ideal 1T-MoSe_2_/SWCNTs were favorable for boosting Li^+^ storage, displaying a distinct synergetic effect between the MoSe_2_ nanoflakes and SWCNT. Lian and co-workers reported carbon-coated 2D SnS/SnO_2_ nanoflake heterostructures wrapped on carbon nanofibers, with a large surface that increased the electrode–electrolyte contact area and accelerated Li^+^ transport between the electrolyte and active materials [101]. The carbon-coated SnS/SnO_2_ accelerated the diffusion of electrons and also alleviated the volume effect caused by the insertion and extraction of lithium ions when used as the anode for a lithium-ion battery, exhibiting excellent stability (1265.3 mAh g^−1^ at 0.2 A g^−1^) and high-rate capability (398.1 mAh g^−1^ at 2 A g^−1^). Wang and co-workers reported ultrathin MoS_2_ nanoflakes grown on N-doped carbon (NC)-coated TiO_2_ nanotubes to achieve TiO_2_@NC@MoS_2_ tubular nanostructures (Fig. 7) [102]. Specifically, the overall 1D coaxial nanostructures with a middle layer of NC could provide an electron/ion pathway for charge storage and delivery.

Moreover, the hierarchical tubular structures constructed of nanosized subunits can effectively alleviate the volume stress related to the electrochemical reactions. The rate capability of the TiO_2_@NC@MoS_2_ electrode was investigated through galvanostatic measurements at various current densities. The average specific discharge capacities were ~ 925, 855, 756, 670, and 612 mAh g^−1^ at the current densities of 0.1, 0.2, 0.5, 1.0, and 2.0 A g^−1^, respectively. When the current density was again reduced to 0.1 A g^−1^, the capacity of the TiO_2_@NC@MoS_2_ electrode quickly returned to 955 mAh g^−1^, revealing the good reversibility of the electrode materials. The cycling performance of the TiO_2_@NC@MoS_2_ electrode was evaluated at a high current density of 1.0 A g^−1^. From the second cycle onward, the discharge capacity quickly stabilized at around 680 mAh g^−1^. After relatively slow fading, a high reversible capacity of 590 mAh g^−1^ was retained after 200 cycles. Importantly, the Coulombic efficiency was nearly 100%, except in the first few cycles.

### Transition Metal-Based Nanoflakes on 2D Skeleton

**Fig. 8:**
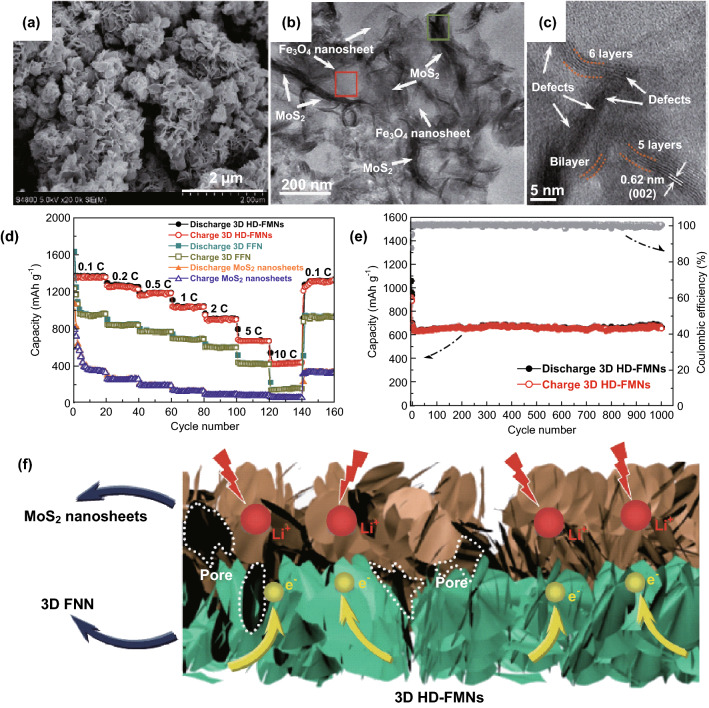
3D architectures based on 2D Fe_3_O_4_/MoS_2_ nanoflakes for LIBs. **a** SEM and **b** TEM image of 3D hierarchical dual Fe_3_O_4_/MoS_2_ nanoflakes. **c** HRTEM image of the MoS_2_ nanoflakes in 3D hierarchical dual Fe_3_O_4_/MoS_2_ nanoflakes. **d** Rate performance of MoS_2_ nanoflakes, 3D Fe_3_O_4_/MoS_2_ nanoflakes, and 3D hierarchical dual Fe_3_O_4_/MoS_2_ electrodes at various current rates. **e** Long-life cycling performance of 3D hierarchical dual Fe_3_O_4_/MoS_2_ nanoflake electrode at 5 C for 1000 cycles. Reproduced with permission [103]. Copyright ©2017, John Wiley & Sons, Inc.

Owing to the unique nanoflake nanostructures, 2D inorganic materials are considered as promising candidates for energy storage. Lu et al. [103] prepared a 3D hierarchical dual Fe_3_O_4_/MoS_2_ (HD-FMN) hybrid nanoarchitecture with an ideal network that was highly effective for improving the performance of LIBs, where the unique structure could provide more contact between MoS_2_ and the Fe_3_O_4_ nanoflakes (Fig. 8). The majority of MoS_2_/Fe_3_O_4_ hybrids are effective for circumventing the key challenges of MoS_2_-based anode materials for LIBs because: (1) the 2D MoS_2_ nanoflakes are tightly anchored in the highly flexible 3D Fe_3_O_4_ nanoflake network and can endure the volume change of the active electrode during lithium insertion/extraction and effectively suppress restacking and exfoliation of the MoS_2_ nanoflakes. Importantly, the intimate surface-to-surface contact between highly crystalline Fe_3_O_4_ and the MoS_2_ nanoflakes can ensure fast electrochemical reaction kinetics, especially at high current rate, which leads to enhanced rate capability and superior stability. (2) As in the case of the 3D HD-FMN electrode with integrative characteristics, high surface area, and a 3D conductive network, electron and ion transport into the deep sites of the entire electrode can be effectively facilitated. (3) The adequate micropores and mesopores should offer sufficient void space for expansion and contraction of the active materials during the lithiation/delithiation process and hence relieve the mechanical stress on the electrode. (4) The ultrathin Fe_3_O_4_ and MoS_2_ nanoflakes with few-layered and defect-rich structures may supply sufficient active sites for hosting the lithium ions and thus effectively shorten the diffusion path for both electrons and ions. The prepared 3D hierarchical dual Fe_3_O_4_/MoS_2_ nanoflakes displayed a maximum reversible specific capacity of 1355 mAh g^−1^ at 0.1 C with outstanding rate capability (698 mAh g^−1^ at 5 C) and long cycle life (650 mAh g^−1^ over 1000 cycles at 5 C), evidencing their great potential as anode materials for LIBs. Besides being promising candidates as high-performance LIB anodes, it is believed that 3D HD-FMNs also have potential applications in other energy storage and conversion fields, such as supercapacitors, batteries, and catalysis.

### Transition Metal-Based Nanoflakes on 3D Skeleton

**Fig. 9:**
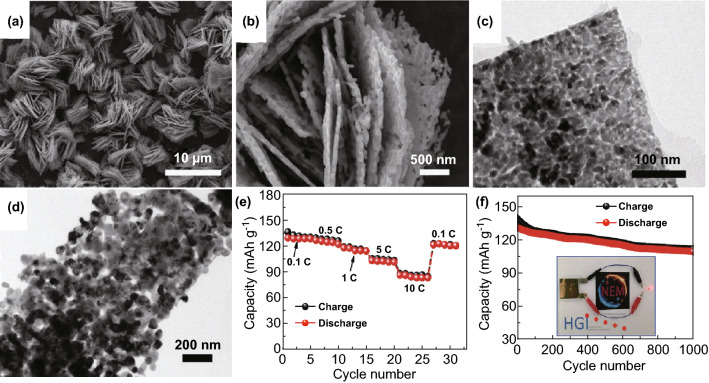
3D architectures based on 2D nanoflake arrays on 3D skeleton ensembles for LIBs. **a, b** FESEM images of layered-LiCoO_2_ nanoflake arrays. **c** TEM image of a single Co_3_O_4_ nanoflake. **d** TEM image of a single-layered LiCoO_2_ nanoflake. **e** Rate performance of LiCoO_2_//Li_4_Ti_5_O_12_ full cell. **f** Cycle performance of LiCoO_2_//Li_4_Ti_5_O_12_ full cell (inset photograph shows the full cell device lighting a LED). Reproduced with permission [104]. Copyright ©2018, John Wiley & Sons, Inc.

Extensive efforts have been devoted to fabricating 3D self-standing nanoarray electrodes on various flexible substrates such as carbon cloth and metal foils. Xue et al. [104] prepared porous LiCoO_2_ nanoflake array assemblies of templated Au-coated stainless steel by a facile “hydrothermal lithiation” method and thermal treatment (Fig. 9). First, the nanoflake arrays were directly grown on the metal substrates, enabling good adhesion and electrical contact, thus leading to fast electron transport between the electrode and substrate and good flexibility of the electrode. Second, the 3D configuration of the electrode could facilitate electrolyte penetration and provide good accommodation of the strain associated with volume change during lithiation/delithiation, resulting in fast ion transport and good mechanical stability. Third, the mesoporous structure and small particle size of the nanoflakes significantly shortened the Li^+^ diffusion length and increased the surface area, thus realizing high utilization of the active material and outstanding rate performance. The prepared electrode displayed excellent lithium storage performance, with outstanding reversible capacity (136 mAh g^−1^), superior rate capability (104.6 mAh g^−1^ at 10 C), and exceptional cycling stability (1.82% decay per cycle over 1000 cycles). This combined level of performance can be attributed to the well-designed interface interaction, nanostructure, and microstructure.

**Fig. 10:**
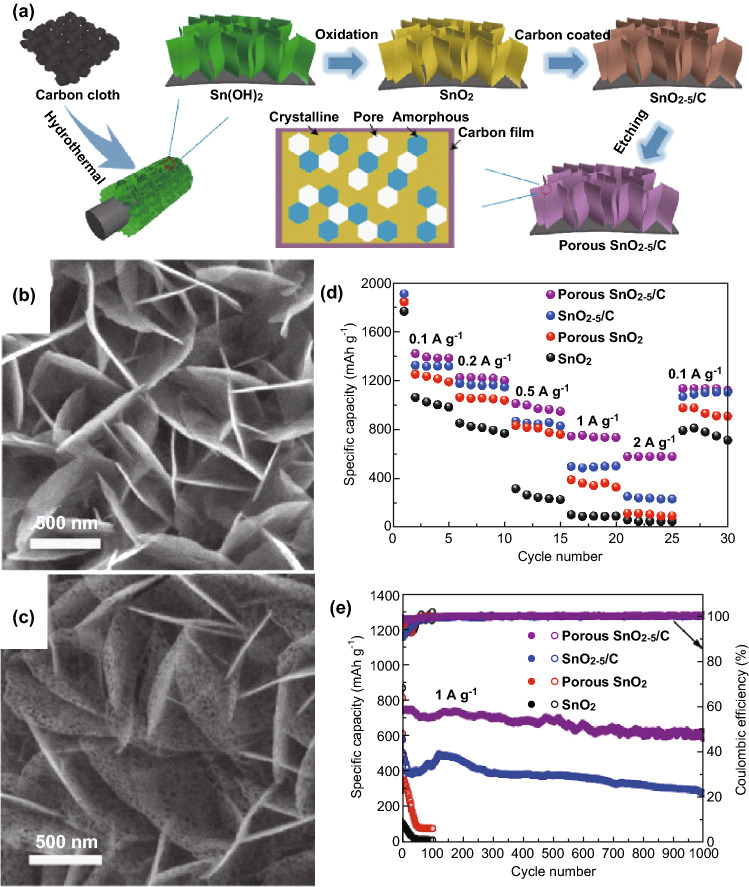
3D architectures based on 2D porous SnO_2-δ_/C nanoflake arrays on carbon cloth substrate ensembles for LIBs. **a** Schematic diagram of the process for fabrication of porous SnO_2-δ_/C nanoflake arrays. **b** SEM image of porous SnO_2-δ_/C nanoflake arrays. **c** SEM image of porous SnO_2_ nanoflake arrays. **d** Rate performance of porous SnO_2-δ_/C, SnO_2-δ_/C, porous SnO_2_, and SnO_2_ nanoflake array electrodes. **e** Cycle performance of porous SnO_2-δ_/C, SnO_2-δ_/C, porous SnO_2_, and SnO_2_ nanoflake array electrodes. Reproduced with permission [105]. Copyright ©2018, Elsevier

Jia and co-workers reported porous SnO_2-δ_/C nanoflake arrays grown on a carbon cloth substrate, with both crystalline and amorphous domains, by a facile strategy (Fig. 10) [105]. Notably, with this unique hybrid nanoarchitecture, the speed of electron transfer and ion diffusion could be improved, and robust structural cohesion and highly reversible conversion reaction could be simultaneously realized in the porous SnO_2-δ_/C hybrid electrode. The improvements offered by the porous SnO_2-δ_/C nanoflake arrays could overcome the key challenges of SnO_2_-based anode materials for LIBs as follows: (1) the introduction of oxygen vacancies enhances the rate of electron transfer and enhances the kinetics of the electrochemical reaction. (2) The presence of the carbon shell ensures the structural integrity of the electrode material during the lithiation process and improves the conductivity. (3) Due to its stable chemical structure and fast charge transfer and ion diffusion rates, the overall material exhibits excellent electrochemical performance and becomes an ideal anode electrode material for LIBs. The prepared carbon-shelled porous SnO_2-δ_ nanoflake arrays displayed a maximum reversible specific capacity of 1133.2 mAh g^−1^ at 0.1 A g^−1^ with outstanding rate capability (574.1 mAh g^−1^ at 2 A g^−1^), which can be attributed to the rational nanoarchitecture design, favoring fast electron and ion transport. Specific reversible capacities of 272.6 and 543 mAh g^−1^ were retained after 1000 cycles at 1 A g^−1^ for the SnO_2-δ_/C and porous SnO_2-δ_/C nanoflake array electrodes, respectively, indicating that the carbon shell effectively improved the structural stability of the SnO_2_ electrodes during long-term cycling.

As shown above, a well-designed structure, interfacial assembly, and microstructural control are effective for the fabrication of electrode materials with outstanding performance. Table 2 presents a comparison of the electrochemical performance of 2D nanoflake ensemble-based materials as electrodes for LIBs [89, 91, 100, 102–106]. Self-assembly of the 2D nanoflakes always forms flower-like structures. However, the dense accumulation of the nanoflakes exerts adverse effects on the diffusion of ions. The 3D structure of the 2D nanoflakes provides sufficient space for ion diffusion, but with sacrifice of the material density. Therefore, the space generated during self-assembly of the two-dimensional nanoarrays is increased, and the space created by the assembly of other materials is reduced, and the balance between the ion transport and the density can be controlled, leading to improved electrochemical performance. Another solution is to introduce materials with excellent electrochemical energy storage properties. The synergistic effect of proper introduction of other materials can greatly enhance the electrochemical performance of the resulting composites. The key factor in the introduction of other materials is the control of the interaction between the 2D nanoflakes and other materials. This unique 3D structure will increase the rate of electron transport and ion diffusion, ultimately leading to enhanced energy storage.

**Table 2:** Comparison of electrochemical performance of 2D nanoflake ensemble-based materials for lithium-ion batteries

Materials	Rate performance	Cycling performance	Refs.
3D hierarchical MoS_2_/C nanoflowers	975 mAh g^−1^ at 0.1 A g^−1^	888.1 mAh g^−1^ after 50 cycles at 0.1 A g^−1^	[89]
MoS_2_ nanoflakes assembling superstructure	1000 mAh g^−1^ at 0.05 A g^−1^	650 mAh g^−1^ after 500 cycles at 1 A g^−1^	[91]
1T MoSe_2_/SWCNTs	630 mAh g^−1^ at 3 A g^−1^	971 mAh g^−1^ after 100 cycles at 0.3 A g^−1^	[100]
Three-layered TiO_2_@carbon@MoS_2_ hierarchical nanotubes	925 mAh g^−1^ at 0.1 A g^−1^	590 mAh g^−1^ after 200 cycles at 1 A g^−1^	[102]
3D hierarchical dual Fe_3_O_4_/MoS_2_ nanoflakes	1355 mAh g^−1^ at 0.1 A g^−1^	650 mAh g^−1^ after 1000 cycles at 5 A g^−1^	[103]
Porous LiCoO_2_ nanoflake arrays	104.6 mAh g^−1^ at 10 C	110 mAh g^−1^ after 1000 cycles at 0.1 C	[104]
SnO_2-δ_/C composite	1378.6 mAh g^−1^ at 0.1 A g^−1^	543 mAh g^−1^ after 1000 cycles at 1 A g^−1^	[105]
Few-layered MoS_2_ nanoflakes on rGO	1142 mAh g^−1^ at 0.1 A g^−1^	753 mAh g^−1^ after 1000 cycles at 2 A g^−1^	[106]

As the most promising substitute for LIBs, sodium-ion batteries have attracted increasing attention because of the abundance of sodium reserves. More importantly, because the unique design of the 3D structure is the same for sodium-ion batteries, the synthesis and surface modification of novel 2D nanoflakes applied to lithium-ion batteries can be extended to sodium-ion batteries, potassium-ion batteries, and zinc-ion batteries.

## 2D Nanoflake Ensemble-Based Materials for Sodium-Ion Batteries

As a potential replacement for LIBs, sodium-ion batteries (SIBs) have received widespread attention [107–110]. Compared with lithium, sodium has lower economic cost and is thus advantageous for widespread application [111–113]. With the rapid development of LIBs, especially the synthesis of various electrode materials, SIBs have developed accordingly in recent years [114–117]. However, Na^+^ is much larger than Li^+^ (0.106 vs. 0.076 nm), resulting in slow electrochemical reaction kinetics, along with significant volume change of the electrode materials during the charge/discharge process [118–121]. Layered transition metal sulfides, because of their obvious interlayer spacing, can promote the insertion and removal of sodium ions and are regarded as ideal electrode materials [122–127]. Two-dimensional nanoflake ensemble-based nanomaterials have been widely explored as electrode materials for SIBs. In this section, our focus is on the use of 2D nanoflake assembly electrode materials for sodium-ion batteries. The electrochemical performance of 2D nanoflake ensemble-based composites, used as electrodes for SIBs, is compared and summarized in Table 3 [128–135].

**Table 3:** Comparison of electrochemical performance of 2D nanoflake ensemble-based materials for sodium-ion batteries

Materials	Rate performance	Cycling performance	Refs.
Hierarchical VS_2_ nanoflakes assemblies	790 mAh g^−1^ at 0.1 A g^−1^	700 mAh g^−1^ after 100 cycles at 0.1 A g^−1^	[128]
2D MoSe_2_/graphene nanocomposites	432 mAh g^−1^ at 0.1 A g^−1^	324 mAh g^−1^ after 1500 cycles at 3.2 A g^−1^	[129]
MoS_2_ nanoflakes aligned vertically on carbon paper	348 mAh g^−1^ at 0.04 A g^−1^	230 mAh g^−1^ after 100 cycles at 0.08 A g^−1^	[130]
Hierarchical flower-like VS_2_ nanoflakes	600 mAh g^−1^ at 0.1 A g^−1^	352 mAh g^−1^ after 700 cycles at 2 A g^−1^	[131]
Self-supported VG/MoSe_2_/N–C sandwiched arrays	540 mAh g^−1^ at 0.2 A g^−1^	298 mAh g^−1^ after 1000 cycles at 2 A g^−1^	[132]
Carbon-coated hierarchical SnS nanotubes	520 mAh g^−1^ at 0.05 A g^−1^	440.3 mAh g^−1^ after 100 cycles at 0.2 A g^−1^	[133]
MoS_2_/graphene nanoflakes	201 mAh g^−1^ at 50 A g^−1^	441 mAh g^−1^ after 250 cycles at 0.3 A g^−1^	[134]
Metallic 1T MoS_2_ sandwich grown on graphene tube	241 mAh g^−1^ at 0.5 A g^−1^	313 mAh g^−1^ after 200 cycles at 0.05 A g^−1^	[135]

### Transition Metal-Based Nanoflakes

**Fig. 11:**
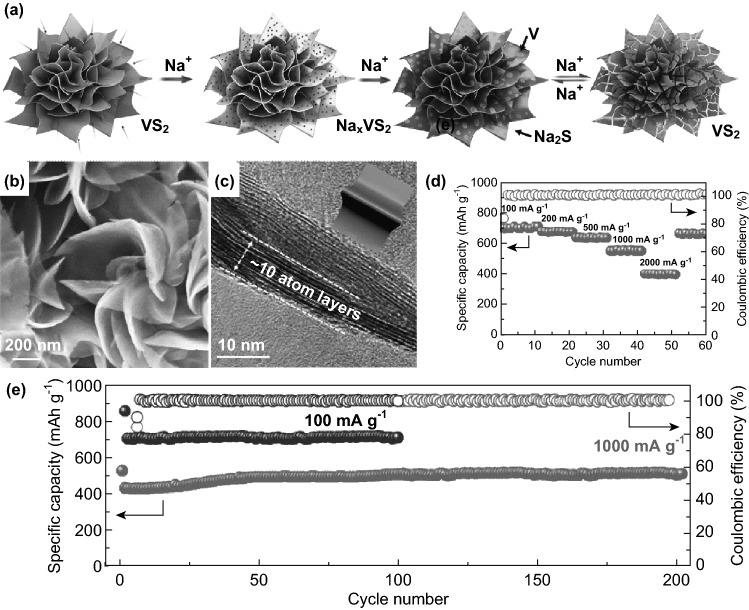
3D architectures based on 2D VS_2_ nanoflake self-assemblies for SIBs. **a** Schematic illustration of reversible Na^+^ ion storage in VS_2_ nanoflake assemblies. **b** SEM images and **c** TEM images of VS_2_ nanoflake assemblies. **d** Rate capability from 100 to 2000 mA g^−1^ and corresponding Coulombic efficiency. **e** Cycling stability and corresponding Coulombic efficiency at 100 mAh g^−1^ and 1000 mA g^−1^. Reproduced with permission [128]. Copyright ©2017, John Wiley & Sons, Inc.

When choosing anode materials for sodium-ion batteries, many transition metal sulfides are potential targets, including VS_2_, MoS_2_, and CoS_2_, because of their high theoretical capacity compared with that of carbon-based systems, and safer working potential versus Na/Na^+^, benefiting from their appealing electrochemical conversion reactions [136]. For example, Zhou et al. [128] prepared hierarchical VS_2_ nanoflake assemblies as a universal electrode material for sodium-ion batteries (Fig. 11). First, the layered 2H VS_2_ nanoflake structure greatly improved the intercalation of Na^+^ ions based on density functional theory calculations. Second, the unique 3D hierarchical structure assemblies comprising numerous ultrathin 2D nanoflakes also played a key role. Moreover, the open space between the nanoflakes within the hierarchical nanoflake assemblies also allowed for easy mass transport and effectively buffered the volume change during repetitive cycling. The initial discharge capacity reached 790 mAh g^−1^, and a discharge capacity of 680 mAh g^−1^ was reversibly recovered and sustained in the ensuing cycles. Moreover, the VS_2_ nanoflakes demonstrated impressive cycling stability. When galvanostatically cycled at 100 mA g^−1^, the specific capacity was maintained at 700 mAh g^−1^ for at least 100 cycles. It was further demonstrated that high-rate cycling of VS_2_ NSA did not compromise its stability (500 mAh g^−1^ after 200 cycles at 1 A g^−1^). Yu et al. [131] reported a facile solvothermal process for fabricating self-assembled hierarchical flower-like VS_2_ nanoflakes, and their Na^+^ storage behavior was systematically studied with respect to the galvanostatic charge/discharge profiles, cyclic voltammograms, rate capability, and long-term cycle stability. Flower-like VS_2_ delivered a high reversible capacity of around 600 mAh g^−1^ at 0.1 A g^−1^ and excellent cycle stability with 83% and 87% retention of the initial capacity after 700 cycles at 2 and 5 A g^−1^, respectively. Moreover, the VS_2_ nanostructures also exhibited superior rate performance with a discharge capacity of 277 mAh g^−1^ at a current density as high as 20 A g^−1^.

### Transition Metal-Based Nanoflakes on 1D Skeleton

Besides VS_2_ nanoflakes, many other 2D TMD nanoflakes, such as MoS_2_ and MoSe_2_, also exhibit superior high-rate performance. A number of 2D TMD nanoflakes containing MoS_2_ have been developed as electrode materials for SIBs. Similar to the case of lithium-ion batteries, 2D TMDs with larger interlayer spacings are key sodium-ion battery materials, where these materials facilitate rapid insertion and removal of sodium ions. For instance, MoS_2_ nanoflakes with different interlayer distances ranging from 0.62 to 0.78 nm were prepared for SIBs [103, 137]. The resultant electrode based on MoS_2_ nanoflakes with the largest interlayer distance exhibited the highest capacity and best rate capability. Moreover, the as-prepared few-layer MoS_2_ anchored on the N-doped carbon ribbon electrode also showed good cycling stability for 300 cycles, with reversible capacities of 495 and 302 mAh g^−1^ at 0.05 and 2 A g^−1^, respectively. Interestingly, the capacity continued to increase during continuous charge/discharge cycles for the MoS_2_ electrodes when cycled at different current densities, and this increase was especially pronounced at 0.5 A g^−1^.

**Fig. 12:**
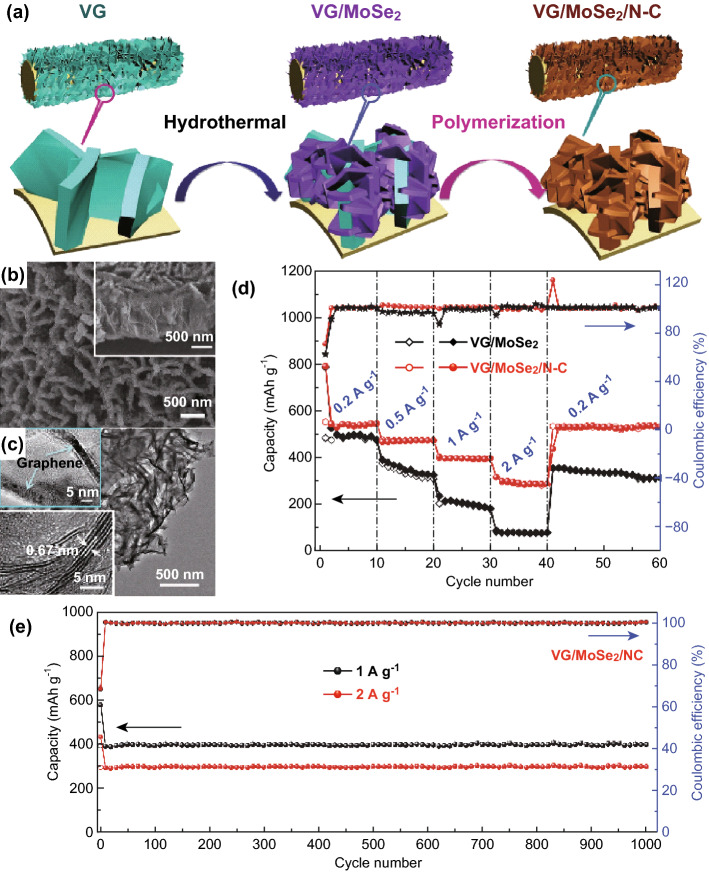
3D architectures based on 2D MoSe_2_ nanoflakes on 1D vertical graphene skeleton for SIBs. **a** Schematic illustration of synthesis of sandwiched VG/MoSe_2_/N–C core/shell arrays. **b** SEM and **c** TEM images of VG/MoSe_2_ arrays. **d** Rate capability and **e** cycling stability of VG/MoSe_2_/N–C electrodes at 1 and 2 A g^−1^, respectively. Reproduced with permission [132]. Copyright ©2016, John Wiley & Sons, Inc.

**Fig. 13:**
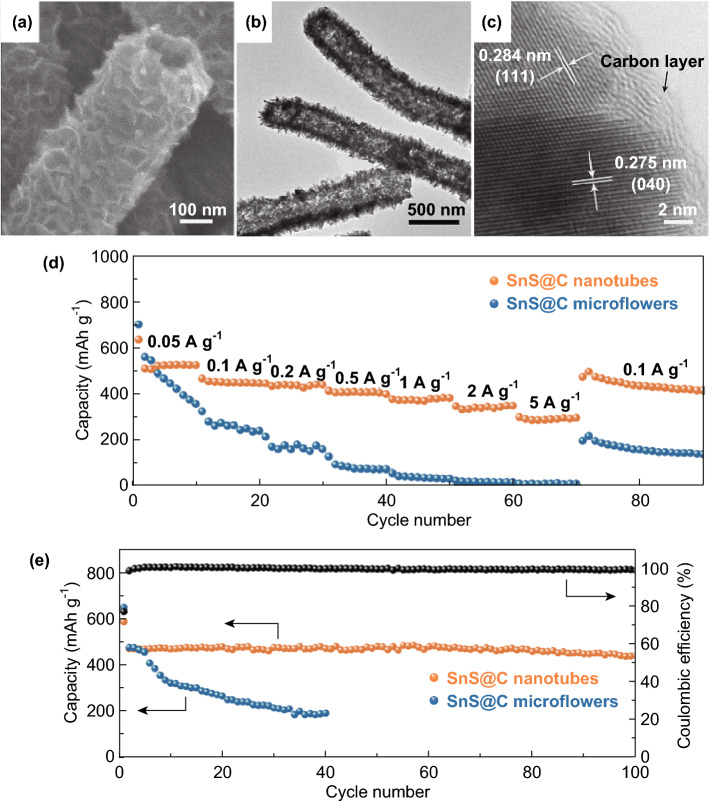
3D architectures based on 2D ultrathin SnS nanoflakes on 1D carbon nanotube skeleton for SIBs. **a** FESEM and **b** TEM images of hierarchical SnS@C nanotubes. **c** HRTEM image of carbon-coated ultrathin SnS nanoflakes. **d** Rate performance of SnS@C nanotubes and microflowers. **e** Cycling performance of SnS@C nanotubes and microflowers at 0.2 A g^−1^, and Coulombic efficiency of SnS@C nanotubes during cycling. Reproduced with permission [133]. Copyright ©2016, John Wiley & Sons, Inc.

Xie et al. reported wrinkled MoSe_2_ nanoflakes sandwiched by a vertical graphene core and N-doped carbon shell, forming sandwiched core/shell arrays. Evaluation of the sodium-ion storage properties displayed high capacity (540 mAh g^−1^), enhanced rate capability, and long-term cycling stability (298 mAh g^−1^ at 2.0 A g^−1^ after 1000 cycles) (Fig. 12) [132]. He et al. [133] reported the design and synthesis of hierarchical nanotubes constructed from ultrathin SnS nanoflakes through a simple and facile templating strategy (Fig. 13). By in situ coating with a carbon precursor and subsequent carbonization, SnS@C nanotubes were easily synthesized. Due to the structural advantages, the SnS@C nanotubes exhibited enhanced sodium storage properties in terms of long cycle life and good rate capability, compared to the SnS@C nanoflowers. Specifically, the SnS@C nanotubes achieved a reversible capacity as high as 440 mAh g^−1^ after 100 cycles at 0.2 A g^−1^. Moreover, at a current density of 5 A g^−1^, the SnS@C nanotubes could still deliver a capacity of 290 mAh g^−1^.

### Transition Metal-Based Nanoflakes on 2D Skeleton

Two-dimensional layered metal sulfides with structures analogous to that of graphite, such as MoS_2_, WS_2_, and VS_2_, have been proposed as a promising family of anode materials for SIBs. Sun and co-workers reported the preparation of a new type of hybrid material containing MoS_2_/graphene nanoflakes prepared by ball milling and exfoliation of commercial bulk MoS_2_ and graphite [134]. The newly prepared MoS_2_/graphene (G) hybrids demonstrated extraordinary rate capability at a high current density of 50 A g^−1^, with a stable reversible capacity of 201 mAh g^−1^. The composite also showed outstanding cycling stability with 95% capacity retention at 0.3 A g^−1^ after 250 cycles.

**Fig. 14:**
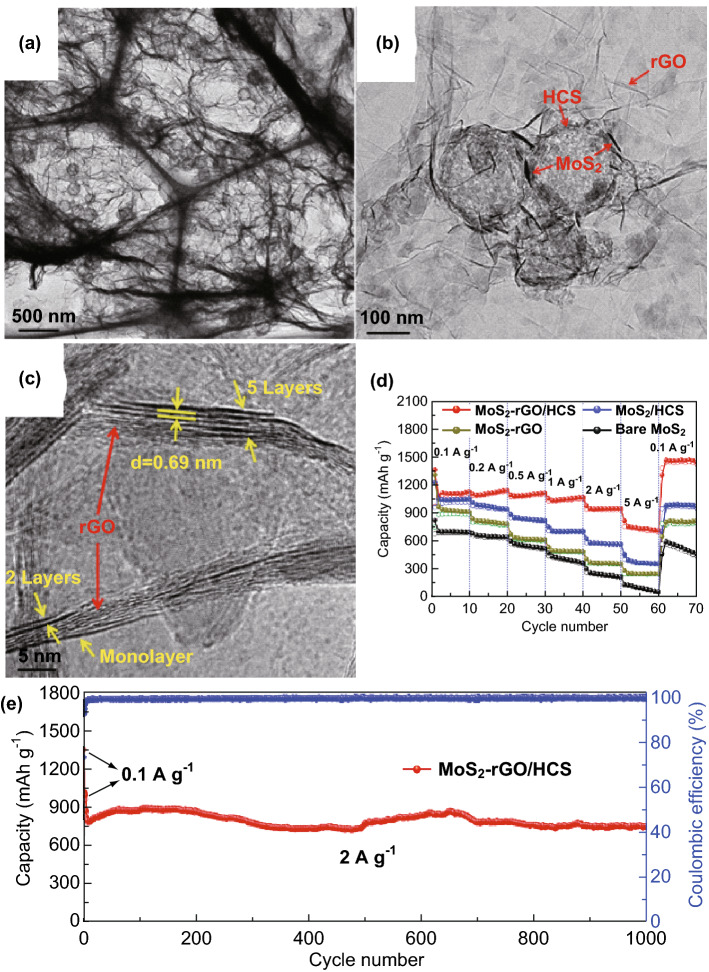
3D architectures based on 2D MoS_2_/rGO nanoflakes for SIBs. **a, b** TEM images, **c** HRTEM image of the MoS_2_-rGO/hollow carbon sphere composite. **d** Rate capability of MoS_2_-rGO/hollow carbon spheres, MoS_2_/hollow carbon spheres, MoS_2_-rGO, and bare MoS_2_. **e** Cycling performance and Coulombic efficiency of MoS_2_-rGO/hollow carbon spheres for 1000 cycles at the current density of 2 A g^−1^. Reproduced with permission [106]. Copyright ©2018, American Chemical Society

Hu et al. [106] reported a facile and reliable strategy for the in situ growth of few-layer MoS_2_ nanoflakes on reduced graphene oxide cross-linked hollow carbon spheres with formation of 3D network nanohybrids (Fig. 14). Notably, this porous structure not only provides enough space to ease volume changes, but also provides a convenient channel for the transport of electrolytes and ions, and affords a continuous conductive network for expedited electron transfer.

MoS_2_-rGO/HCS exhibits attractive electrochemical performance for sodium storage, which can be primarily attributed to the favorable architecture and properties as follows: (1) the ultrathin MoS_2_ nanoflakes tightly overlay the interconnecting rGO/HCS conductive networks, which not only effectively hinders aggregation of the MoS_2_ nanoflakes and restacking of the graphene nanoflakes, but also can cushion the volume change caused by charging and discharging, and is conducive to improving the stability and recyclability of the structure. (2) The 3D porous scaffolds of rGO/HCS are in intimate face-to-face contact; in this way, the few-layer MoS_2_ nanoflakes can greatly enhance the electrical conductivity and facilitate electrolyte/ion transport, resulting in satisfactory electrochemical reaction kinetics, large capacity, and optimal rate performance. (3) The larger interlayer spacing may alleviate the stress of sodium-ion insertion and removal, thereby enhancing the speed of insertion of sodium ions and providing more insertion sites. (4) This unique 3D honeycomb-like network structure can increase the contact area between the active material and the electrolyte, thereby increasing the number of sodium-ion insertion sites and shortening the sodium-ion diffusion pathway. At a current density of 5 A g^−1^, the MoS_2_-rGO/HCS electrode delivered an average discharge capacity of 364 mAh g^−1^.

**Fig. 15:**
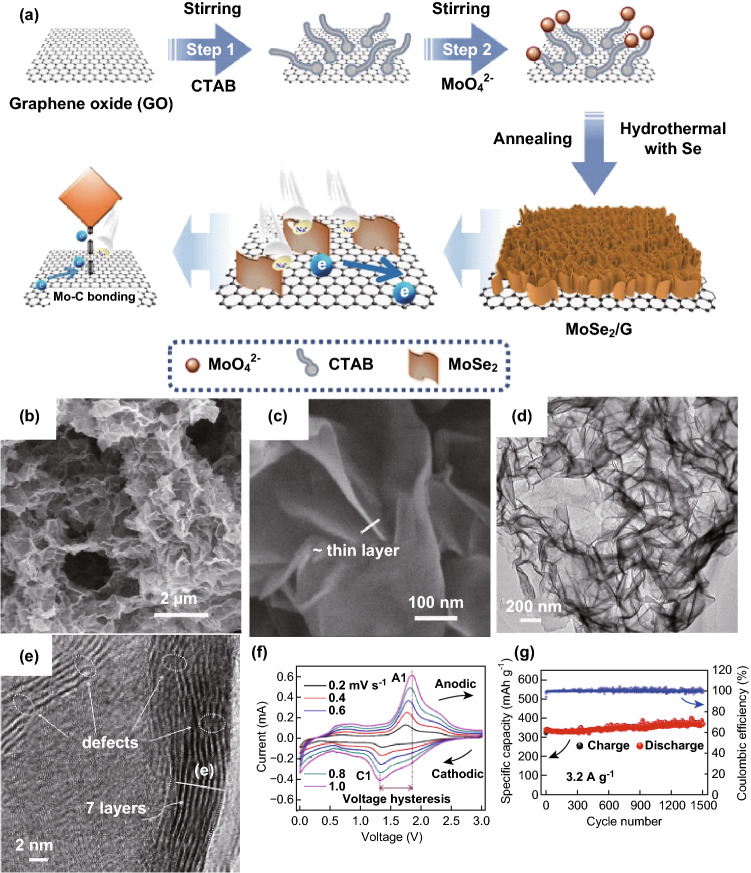
3D architectures based on 2D MoSe_2_/graphene nanoflakes for SIBs. **a** Strategy for fabrication of ordered 2D superstructure. **b, c** SEM images of as-prepared MoSe_2_/G. **d** TEM and **e** HRTEM images of as-prepared MoSe_2_/G. **f** CV curves of MoSe_2_/G superstructure at different scan rates from 0.2 to 1.0 mV s^−1^. **g** Cycling performance and corresponding Coulombic efficiency of MoSe_2_/G superstructures at a current density of 3.2 A g^−1^ for 1500 cycles. Reproduced with permission [129]. Copyright ©2018, Elsevier

To further examine the long-term stability of MoS_2_-rGO/HCS, a cycling test was carried for 500 cycles out with an initial current density of 0.1 A g^−1^ for the first three cycles and a current density of 1 A g^−1^ for the following hundreds of cycles. After 500 cycles, a high reversible capacity of 443 mAh g^−1^ was maintained, with a Coulombic efficiency of around 100%, reconfirming the outstanding cycling stability of this material. Zhao and co-workers reported MoSe_2_ nanoflakes perpendicularly grown on graphene, synthesized through a CTAB-assisted hydrothermal method (Fig. 15) [129]. The few-layered, highly defective MoSe_2_ nanoflake arrays with expanded interlayer space grew perpendicularly on the surface of the graphene nanoflakes, with the formation of Mo-C bonds at the MoSe_2_ and graphene interface. MoSe_2_/G delivered a high reversible capacity of around 324 mAh g^−1^ at 3.2 A g^−1^. The MoSe_2_/G anode demonstrated excellent cycling stability and reversibility. After 1500 cycles, a reversible capacity as high as 368 mAh g^−1^ was retained.

### Transition Metal-Based Nanoflakes on 3D Skeleton

**Fig. 16:**
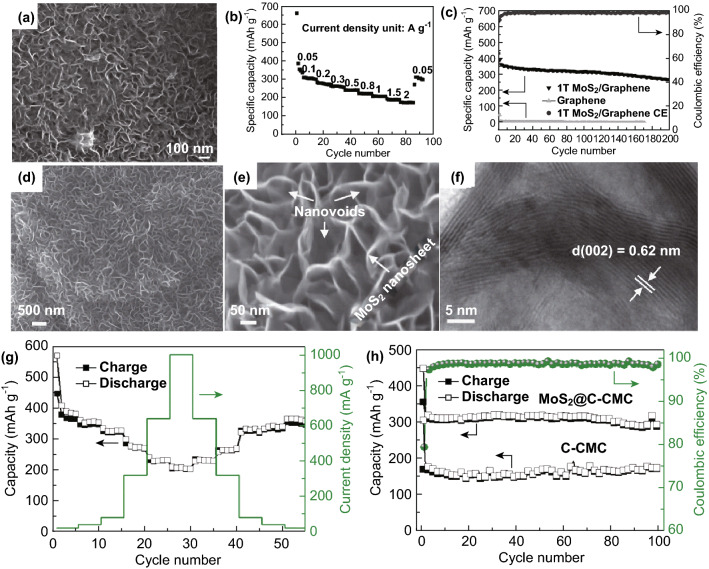
3D architectures based on 2D transition metal nanoflakes on 3D skeleton ensembles for SIBs. **a** High-magnification image of 1T-MoS_2_ on the internal surface of the tube. **b** Rate performance of the MoS_2_-graphene electrode. **c** Specific capacity and Coulombic efficiency versus cycle number at a current density of 50 mA g^−1^. Reproduced with permission [135]. Copyright ©2017, John Wiley & Sons, Inc. **d, e** SEM images of the as-prepared freestanding MoS_2_@C. **f** High-magnification TEM image of MoS_2_@C. **g** Rate performance of MoS_2_@C-CMC electrode. **h** Cycling performance and corresponding Coulombic efficiency of MoS_2_@C-CMC paper electrode at 80 mA g^−1^ and cycling performance of CMC-coated carbon paper electrode. Reproduced with permission [130]. Copyright ©2016, John Wiley & Sons, Inc.

Due to the low conductivity and the huge volume variations of transition metal nanoflakes during charge/discharge processes, bare transition metal nanoflake electrodes exhibit poor rate capability and fast capacity decay upon cycling. Generally, current collectors, conductive agents, and binders are needed to fabricate film electrodes, which inherently increase the total weight and cost of SIBs. Xie and co-workers reported the deposition of MoS_2_ nanoflakes on carbon derived from paper towels for SIBs, where the hierarchical structure enabled sufficient electrode/electrolyte interaction and fast electron transport (Fig. 16a–c) [130]. Moreover, the unique architecture could minimize the excessive interface between carbon and the electrolyte, leading to a high initial Coulombic efficiency. The high surface-to-volume ratio of the nested structure with nanoreservoirs between adjacent MoS_2_ nanoflakes promoted interaction between MoS_2_ and the electrolyte. The MoS_2_ layers coated on the carbon fibers diminished the carbon–electrolyte interaction and reduced the side reactions responsible for the irreversible capacity. In the coaxial structure, the MoS_2_ nanoflakes were connected to the conductive carbon fibers, thus affording good current collector/active material electrical contacts and low charge transfer resistance. Moreover, the MoS_2_ nanoflakes were vertically aligned on the carbon paper matrix in a manner that concurrently constructed efficient ion and electron transfer pathways, overcoming the kinetic limitations. This phenomenon indicates that the interlayer spacing of MoS_2_ was gradually expanded during cycling, and thus more active sites were generated for the electrochemical reaction, and the energy barrier for intercalation/de-intercalation of the sodium ions was further decreased. For example, the metallic 1T-MoS_2_ sandwich grown on a graphene tube delivered a high reversible capacity of 313 mAh g^−1^ at a current density of 0.05 A g^−1^ after 200 cycles and a high-rate capability of 175 mAh g^−1^ at 2 A g^−1^ (Fig. 16d–h) [135]. Obviously, at the current stage, the performance of 2D TMD nanoflakes for SIBs is still not comparable to that for LIBs in terms of the specific capacity, rate capability, and cycling performance.

## Other Applications

In addition to the aforementioned applications, other kinds of potential applications of these 3D architectures have also been demonstrated, such as potassium-ion batteries and zinc-ion batteries.

### 2D Nanoflake Ensemble-Based Materials for Potassium-Ion Batteries

Along with the rapid development of LIBs and SIBs, potassium-ion batteries (KIBs) have also become an attractive alternative to LIBs or SIBs, owing to the abundant natural resources and similar chemical/physical properties of K to Li or Na [138–142]. More importantly, K has a lower standard redox potential than Na (and even Li) in non-aqueous electrolytes, which can be translated into a potentially higher cell voltage of KIBs compared with those of SIBs and LIBs [143–147]. Okoshi et al. [148] showed that K electrolytes exhibit higher conductivity than Li and Na electrolytes. Given these advantages, KIBs have rapidly attracted considerable interest, and various materials have been developed and evaluated as potential KIB electrodes.

**Fig. 17:**
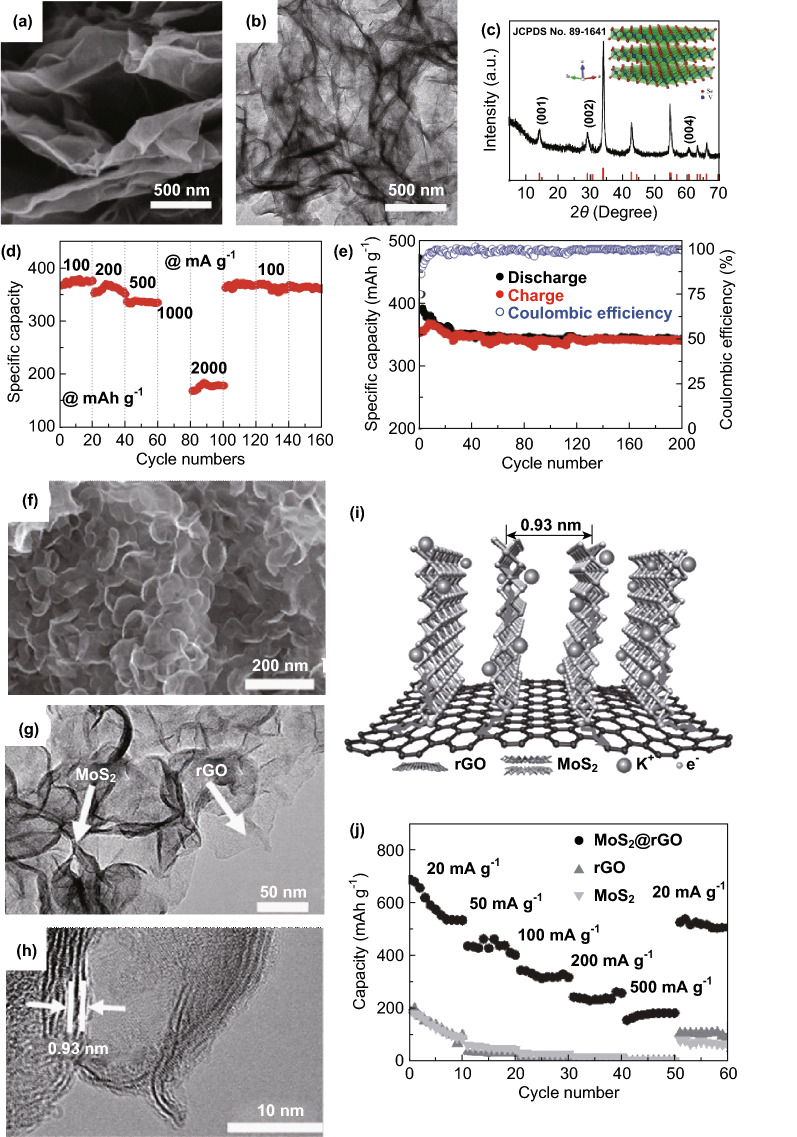
2D nanoflake ensemble-based materials for KIBs. **a** SEM, **b** TEM, and **c** XRD pattern of ultrathin layered VSe_2_ nanoflakes. **d** Rate capabilities of VSe_2_ nanoflakes. **e** Cycling stability and Coulombic efficiency of VSe_2_ nanoflake electrode at 0.2 A g^−1^. Reproduced with permission [149]. Copyright ©2018, John Wiley & Sons, Inc. **f** SEM, **g** TEM, and **h** HRTEM images of MoS_2_@rGO composite. **i** Schematic illustration showing paths for K-ion diffusion and electron conduction in MoS_2_@rGO composites. **j** Rate performance of MoS_2_@rGO, MoS_2_ particle, and pure rGO. Reproduced with permission [150]. Copyright ©2017, John Wiley & Sons, Inc.

For example, Guo and co-workers reported single-crystalline metallic graphene-like VSe_2_ ultrathin nanoflakes as anode materials for enhancing the capacity, rate ability, and cycling stability of KIBs (Fig. 17a–e) [149]. The large-sized ultrathin wrinkled-like nanoflakes are considered as an ideal architecture for high-performance KIBs due to their large surface area-to-volume ratio, ultrafast electron/K-ion transport, restricted self-aggregation, and superior structural stability. Benefitting from the unique 2D nanostructure, the ultrathin VSe_2_ nanoflakes exhibit very high reversible capacity, high-rate capability, and very low decay, making them the best among anode materials reported for KIBs. Xie et al. [150] developed a new two-step solvothermal strategy for the synthesis of ultrathin and ultrauniform rose-like MoS_2_ nanoflakes strongly coupled with rGO nanoflakes (Fig. 17f–i). These MoS_2_@rGO composites, with an expanded MoS_2_ interlayer spacing, comprising chemically bonded MoS_2_ and rGO with a unique nanoarchitecture, displayed the best electrochemical performance relative to related materials. Specifically, (1) expanding the interlayer spacing of layered metal dichalcogenides themselves favorably alleviates the structural change upon ion insertion/extraction [151–153]. (2) Strong coupling between these materials and the highly conductive skeleton materials is the key to enhancing the electrode kinetics, while concurrently buffering the structural change during cycling [154–156]. (3) Carefully tuning the morphology of these materials on the micro-/nanoscale is also an efficient way to enhance their electrochemical performance [157–159]. Compared to LIBs or even SIBs, the history of the electrochemistry of KIBs is generally short. As expected, the successful strategies for the development of LIBs or SIBs can also be applied to KIBs.

### 2D Nanoflake Ensemble-Based Materials for Zinc-Ion Batteries

**Fig. 18:**
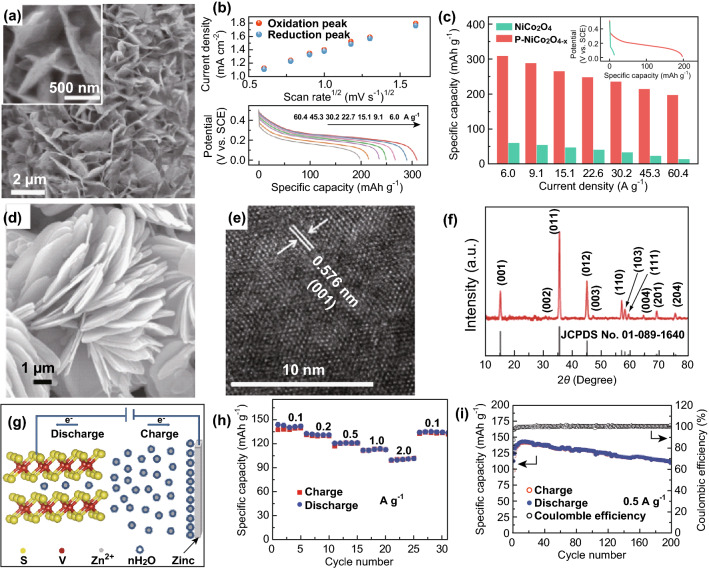
2D nanoflake ensemble-based materials for ZIBs. **a** SEM images of P-NiCo_2_O_4-x_ nanoflakes grown on a carbon cloth substrate. **b** Variation in the redox peak currents with the square root of the scan rate for P-NiCo_2_O_4-x_ electrodes and discharge curves for P-NiCo_2_O_4-x_ electrode at various current densities. **c** Specific capacities of NiCo_2_O_4_ and P-NiCo_2_O_4-x_ electrodes at various current densities. Reproduced with permission [167]. Copyright ©2018, John Wiley & Sons, Inc. Characterization of layered VS_2_: **d** SEM, **e** HRTEM image, and **f** XRD pattern. **g** Schematic illustration of the mechanism of operation of Zn/VS_2_ batteries. **h** Rate capability. **i** Long-term cyclic properties at a current density of 0.5 A g^−1^. Reproduced with permission [168]. Copyright ©2017, John Wiley & Sons, Inc.

Recently, rechargeable zinc-ion batteries (ZIBs) have been recognized as particularly promising because of their safety, low cost, the abundance of Zn sources, and the ability of divalent cations to increase the charge-storage capabilities [160–163]. Many 2D nanoflake materials have been used as active electrode materials for ZIBs [164–166]. In order to meet the ever-increasing energy demand, the preparation of new synthetic electrode materials is imperative. Due to the huge volume change of electrode materials during the charge/discharge process, it is still a challenge to find suitable electrode materials. Zeng et al. [167] introduced oxygen vacancies and phosphate ions into an ultrathin NiCo_2_O_4_ nanoflake ensemble, thereby significantly boosting its electrochemical performance (Fig. 18a–c). Modulation of the oxygen vacancies and surface phosphate ions was achieved by annealing the pristine NiCo_2_O_4_ nanoflakes using a simple phosphating process. Benefiting from the merits of the substantially improved electrical conductivity and increased concentration of active sites, the optimized P-NiCo_2_O_4-x_ nanoflake electrode delivered a high reversible capacity of 309.2 mAh g^−1^ at 6.0 A g^−1^ and extraordinary rate performance, with 64% capacity retention at 60.4 A g^−1^. Mai and co-workers reported the synthesis of new 2D layered VS_2_ nanoflakes that were assembled to construct 3D architectures, and demonstrated the outstanding properties of this material as a cathode material for rechargeable ZIBs (Fig. 18d–i) [168]. Moreover, the battery delivered a high capacity of 190.3 mAh g^−1^ at a current density of 0.05 A g^−1^ and exhibited long-term cyclic stability with 98% capacity retention over 200 cycles. Compared to alkali ion batteries, the history of the electrochemistry of ZIBs is generally short, and the successful strategies applied to the development of LIBs, SIBs, and KIBs can also be extended to ZIBs.

## Conclusions and Outlook

Presently, the development of advanced science and technology is considered the best choice for solving the problems of the energy environment in the twenty-first century. Due to their large surface area and numerous active sites, two-dimensional nanoflake ensemble-based architectures display unprecedented performance in the aforementioned fields. In this review, we outlined recently developed, unique two-dimensional nanoflake ensemble-based architectures for improving energy storage in devices such as supercapacitors, lithium-ion batteries, sodium-ion batteries, potassium-ion batteries, and zinc-ion batteries. The three-dimensional structure composed of two-dimensional nanoflakes can provide more channels for electrons and ions and can also provide better electrode materials for new energy sources.

Irrespective of the potential application, the first target is controlled synthesis of these composites, which requires precise control of the 3D nanostructures, including controlled growth of the layers, pore size, and porosity. Most of these reported 3D architectures have a wide pore size distribution, ranging from one hundred to several hundreds of micrometers. Large pore sizes decrease the mechanical performance of the resulting materials. Thus, single-layered 3D architectures are hardly ever prepared due to their fragile mechanical features and large pore sizes. The preparation of 3D architectures with uniform meso- or micropores and controlled layers is thus a more effective approach. Achieving control of the layer growth and size of the 2D nanoflakes in the assembly process is a key challenge. The second challenge is to further enhance the mechanical and electrical performance of the 3D architectures. Increasing and strengthening the cross-linking between the nanoflakes by enhancing the surface functional moieties and adding cross-linkers will enhance the internanoflake binding and the mechanical and electrical performance. Thus, continued innovative research and development is required to further enhance the performance and applications of 3D architectures.

For energy storage applications, an advanced hybrid nanostructure should generally meet the requirements of having a large specific surface area for reaction and ion exchange, and conductive networks for charge transport, as well as an interface/heterojunction formed by two components for the effective channeling or separation of charge carriers. Among these requirements, much effort has been devoted to increasing the effective surface area of these hybrids. As such, the surface area of 2D nanoflake ensemble structures is generally much larger than the theoretical value for seamless 2D materials, and the former simultaneously supply plenty of active edge sites for various reactions.

In addition, because the material engineering of 3D architectures leads to combined, composite, or hybrid nanomaterials, the original techniques can provide access to a large number of 2D nanoflake-based 3D nanohybrids that integrate the performance of the individual 2D nanomaterials, and can also furnish new collective and synchronic functions. Furthermore, improving the properties of 2D nanomaterials on the nanoscale is a worthwhile undertaking owing to the synergetic chemical coupling effects that can be derived. On this basis, the importance of fundamental understanding of the principles of these synergistic coupling effects should be emphasized. Furthermore, more theoretical investigations on the electronic properties and crystal and surface structures of different 3D architectures, as well as the synergetic effects of modified 2D nanoflake ensemble-based nanomaterials, should be performed based on first-principle calculations, which, in combination with smart experimental strategies, will greatly expedite the development of extremely efficient 2D nanoflake ensemble-based 3D nanocomposites for energy conversion and storage and other applications.

In short, the large-scale production problems must be resolved. Nanomaterial science is a cross-discipline field. The two-dimensional nanoflakes prepared in different fields have different applications, and assembling two-dimensional nanoflakes into a three-dimensional structure undoubtedly produces unexpected effects. The success of nanotechnology depends on the close cooperation between researchers in different disciplines in order to understand current and future needs. It is necessary to choose suitable methods to prepare two-dimensional nanoflakes and then assemble them or compound them with other materials. The development of new two-dimensional nanoplates for assembly is still in the ongoing trial phase, and it is believed that groundbreaking progress in these technologies will continue to be achieved with the efforts of researchers. Practically, however, basic research and real-life application may take decades to establish.
